# Comparison of preprocessing methods and storage times for touch DNA samples

**DOI:** 10.3325/cmj.2017.58.4

**Published:** 2017-02

**Authors:** Hui Dong, Jing Wang, Tao Zhang, Jian-ye Ge, Ying-qiang Dong, Qi-fan Sun, Chao Liu, Cai-xia Li

**Affiliations:** 1 Key Laboratory of Forensic Genetics, Beijing Engineering Research Center of Crime Scene Evidence Examination, Institute of Forensic Science, Ministry of Public Security, Beijing, China; 2 Guangzhou Forensic Science Institute, Guangdong Province Key Laboratory of Forensic Genetics, Guangzhou, China; 3 Department of Forensic Medicine, Southern Medical University, Guangzhou, China; 4 Human Identification Division, Thermo Fisher Scientific, South San Francisco, CA, USA

## Abstract

**Aim:**

To select appropriate preprocessing methods for different substrates by comparing the effects of four different preprocessing methods on touch DNA samples and to determine the effect of various storage times on the results of touch DNA sample analysis.

**Method:**

Hand touch DNA samples were used to investigate the detection and inspection results of DNA on different substrates. Four preprocessing methods, including the direct cutting method, stubbing procedure, double swab technique, and vacuum cleaner method, were used in this study. DNA was extracted from mock samples with four different preprocessing methods. The best preprocess protocol determined from the study was further used to compare performance after various storage times. DNA extracted from all samples was quantified and amplified using standard procedures.

**Results:**

The amounts of DNA and the number of alleles detected on the porous substrates were greater than those on the non-porous substrates. The performances of the four preprocessing methods varied with different substrates. The direct cutting method displayed advantages for porous substrates, and the vacuum cleaner method was advantageous for non-porous substrates. No significant degradation trend was observed as the storage times increased.

**Conclusion:**

Different substrates require the use of different preprocessing method in order to obtain the highest DNA amount and allele number from touch DNA samples. This study provides a theoretical basis for explorations of touch DNA samples and may be used as a reference when dealing with touch DNA samples in case work.

Skin or mucosal cells can be left on items such as a knife handle, key, socks, or toothbrush at crime scenes, which is known as touch DNA evidence (1). The number of cases with touch DNA samples in forensic investigations has greatly increased since the first demonstration of touch DNA evidence in 1997 (1-3). It is difficult to obtain full DNA profiles from such samples, and repeated amplifications are usually needed, which may slow down the entire process. It is also difficult to locate cells or DNA on the collected evidence, and thus, such evidence is usually processed blindly after being cut into many small samples. If too many samples are cut from a single piece of evidence, the DNA concentration can be decreased, and contamination is a common issue.

Various studies of touch DNA samples were performed, such as experiments on cell-free DNA (4,5), DNA transfer (6,7), the influence of an individual’s age (8), and preprocessing methods (9-17). For the preprocessing method, the use of adhesive tape (9-13) and the double swab technique (14-16) were studied, and cutting the samples into small pieces as one preprocessing method is commonly used in all cases (17). There were studies using the vacuum cleaner method (18,19) when dealing with gunshot residues, but in China, a vacuum cleaner method is used as a preprocessing method to collect cast-off cells on touch samples before DNA extraction.

Some studies compared the differences between two preprocessing methods. For instance, de Bruin et al (20) found that the stubbing method displays better performance over the double swab technique from a practical point of view. Hansson et al (21) compared DNA collection with mini-tape vs three different swabs. There are also studies on the persistence of saliva (22), fingernails (23), biological samples (24), trace evidence (25), and the persistence of DNA in touch samples. However, no comparison has been made for the effects of four different preprocessing methods on touch DNA samples.

Our study had two aims. The first aim was to select appropriate preprocessing methods when dealing with different substrates through the comparison of the effects of four different preprocessing methods on touch DNA samples. The second one was to determine the effect of various storage times on the results of touch DNA sample analysis.

## MATERIAL AND METHODS

We designed two series of experiments to investigate the performance of various preprocessing methods and storage times for touch DNA samples. In the series A of experiments, we investigated four preprocessing methods for touch DNA samples (Table 1). We selected seven common and typical substrates (cloth, thick rope, thin rope, plastic rope, door handle, glove, and glass) as sources of DNA samples. The porous substrates included cloth, gloves, thick cotton ropes, and thin cotton ropes, whereas the non-porous substrates included door handles, glass, and plastic ropes (Table 2). In the series B of experiments, we investigated the effects of various storage times on touch DNA samples. Under laboratory conditions, we used as long a period of time as reasonable and possible to store the mock samples. According to the experimental findings in Series A, the preprocessing method yielding the maximum amount of extracted DNA was chosen in these experiments. Two substrates (gloves and door handles) were investigated after storage for 2, 6, 10, 30, 60, 90, 180, or 360 days.

**Table 1:** Four preprocessing methods used for preparation of substrates for touch DNA sampling

Preprocessing method	Tools*	Tool characteristics	Process	Treatment before DNA extraction
Direct cutting method	Scissors	Sterile	Cutting samples into small pieces	-
Double swab technique	Swabs	Cotton Sterile Tip diameter:^†^ 5 mm Tip length:^‡^ 13 mm	The first swab was moistened with sterile distilled water and used to scrub the entire surface of the samples at an angle of 5-10° in horizontal lines; the second swab was used in the same way but was not moistened. The two swabs were co-extracted.	Air dried for 12 h
Stubbing procedure	EZ-tape	Diameter of the tape: 12 mm	The stub was placed on the surface of the samples and removed until all of the area was covered. Each stub was placed on the surface of the sample approximately 20 times before it was saturated. When the stub no longer adhered to the sample, a second stub was used. When using multiple stubs on one sample, they were co-extracted.	In a dark room
Vacuum cleaner method^§^	Vacuum cleaner	4.84 π-cm^2^ one-off tips	A 25-cm^2^ white impervious membrane was placed between the one-off tip and the tip of a vacuum cleaner. Each sample was vacuumed for approximately 5 s on all surfaces. The 4 π-cm^2^ membrane on which the cast-off cells may have been absorbed was cut off from the 25-cm^2^ membrane waiting for further process.	In a dark room

*Institute of Forensic Sciences, Ministry of Public Security, China.

†Diameter at widest point.

‡Length at longest point to the end of the tip material on the shaft.

§The tip of the vacuum cleaner was a tube. The pipe diameter was 2 cm, and the length was 110 cm. The pipe diameter and the length of the one-off tip, respectively, were 2.2 and 15 cm. We put the 25-cm^2^ membrane between the one-off tip and the tip of a vacuum cleaner, and the one-off tip overlapped the tip of the vacuum cleaner by 0.5 cm. The cast-off cells may be absorbed on the membrane surface of 4π-cm^2^.

**Table 2:** Seven types of substrates (all produced in Beijing, China) used for the preparation of samples for preprocessing method comparison

			Substrate characteristics	
**Substrate type**	material		color	size
Porous				
cloth		weave cotton fiber	white	4.0 × 4.0 cm
glove		weave cotton fiber	white	L
thick rope		weave cotton fiber	white	diameter: 1.0 cm length: 9.0 cm
thin rope		weave cotton fiber	white	diameter: 0.5 cm length: 9.0 cm
Non-porous				
plastic rope		polypropylene	transparent colorless	diameter: 0.5 cm length: 12.0 cm
glass		silicon dioxide	transparent colorless	2.0 × 7.5 cm
door handle		painted wood	brown	diameter: 2.5 cm length: 8.0 cm

### Quality control

Before the experiments, five seal-lock plastic bags, five pieces of clothing, two thick cotton ropes, and two thin cotton ropes were all cut into small pieces using tools manufactured by the Institute of Forensic Sciences, Ministry of Public Security (IFS, MPSC), China. Two plastic ropes were processed using the vacuum cleaner method and three gloves were stubbed, as described in Table 1. The double swab technique was performed on three door handles and five pieces of glass. After DNA extraction, none of these 27 negative controls tested provided detectable DNA or profiles. Both positive and negative controls were implemented for all reactions during the DNA processing steps. In all experiments, analysts were not used either as DNA donors or participants. The ambient temperature in the laboratory was maintained between 22 and 24°C, with a mean relative humidity of 50% during the experiments.

### Experimental set-up

The series A of experiments involved six men and two women, whereas the series B involved four men and one woman (25-30 years old) to create mock touch DNA samples. Each individual was asked not to wash their hands 1 h prior to the experiment and rubbed their hands for 10 s before participating. An internal lag of at least 24 h was performed between the preparations of each touch DNA sample.

The samples were prepared for the series A of experiments (Table 3). In total, 176 samples were generated for further processing. All samples were dried for at least 24 h before conducting the preprocessing methods. For the series B of experiments, each individual was asked to put the gloves on both hands and vigorously rub them for approximately 30 s. This process was repeated for eight gloves per individual. Each glove was placed into a seal-lock plastic bag for storage for various amount of time. Each individual held the door handle and rubbed it vigorously using both hands for approximately 30 s. In total, 80 samples were generated for further processing. All samples were dried for at least 24 h before being placed into the bags.

**Table 3:** Detailed process of preparing touch DNA samples

Substrate	Action*	Treatment after action	Repeat	Number^†^	Preprocessing method used	Final number^‡^
Cloth	Rub	Cut into three equal small pieces (2 × 2cm)	-	3 (small one)	Direct cutting method Double swab technique Stubbing procedure	24
Glove	Rub	-	Three times	3	Direct cutting method Vacuum cleaner method Stubbing procedure	24
Thick rope	Rub	Cut into three equal small pieces (length: 3 cm)	-	3 (small one)	Direct cutting method Vacuum cleaner method Stubbing procedure	24
Thin rope	Rub	Cut into three equal small pieces (length: 3 cm)	-	3 (small one)	Direct cutting method Vacuum cleaner method Stubbing procedure	24
Plastic rope	Rub	Cut into four equal small pieces (length: 3 cm)	-	4 (small one)	Direct cutting method Vacuum cleaner method Stubbing procedure Double swab technique	32
Glass	Press	-	Three times	3	Double swab technique Stubbing procedure Vacuum cleaner method	24
Door handle	Rub	-	Three times	3	Double swab technique Stubbing procedure Vacuum cleaner method	24

*Each action lasted 10 seconds.

†Number of samples used from one individual.

‡Number of samples used from the eight individuals.

### Sample processing and analysis

Different samples were preprocessed with different preprocessing methods for the series A of experiments (Table 3). For the series B, the stubbing procedure was used on the gloves and door handles. Briefly, DNA was extracted using a Mag Attract^®^ M 48 DNA Manual kit (Qiagen, Hilden, Germany) (26), quantified using a Quantifiler^TM^ Human DNA quantification kit (Applied Biosystems, Foster City, CA, USA) and 7500 Real Time PCR system (Applied Biosystems), and genotyped using an AmpFLSTR^®^ Identifiler^®^Plus kit (Applied Biosystems), ABI 3130XL Genetic Analyzer (Applied Biosystems), and Gene Mapper^TM^ ID Software (Applied Biosystems) according to the manufacturers’ protocols. DNA was processed using the procedures identical to those used for case work samples. A calling threshold of 50 relative fluorescence units (RFUs) was used for analysis.

The total amount of DNA rather than the DNA concentration was used for comparisons to explain why full DNA profiles were not always observed when the quantity of DNA recovered was high (>1 ng).The preprocessing methods and storage times were compared and discussed regarding the amount of DNA and the number of alleles detected. Homozygous alleles were counted as two alleles in the analysis. Fifteen short tandem repeat loci were used for analysis, excluding Amelogenin.

To compare the differences among the four preprocessing methods, we separately analyzed the data for the porous and non-porous substrates. Each pair of preprocessing methods was analyzed, and the differences in the amounts of DNA and number of detected alleles among each pair were determined.

### Statistical analysis

All results are presented as the total DNA (mean ± standard deviation [SD]) rather than the DNA concentration. The Student’s *t*-test were used to analyze the data of comparison between the mean amount of DNA of porous substrates and non-porous substrates, and the one-way analysis of variance were used to analyze all data except the above data, with *P* < 0.05 considered significant (27,28). All statistical tests were performed using the SPSS software (IBM SPSS Statistics 19.0, IBM, Armonk, New York).

## RESULTS

### Series A

**Figure 1:**
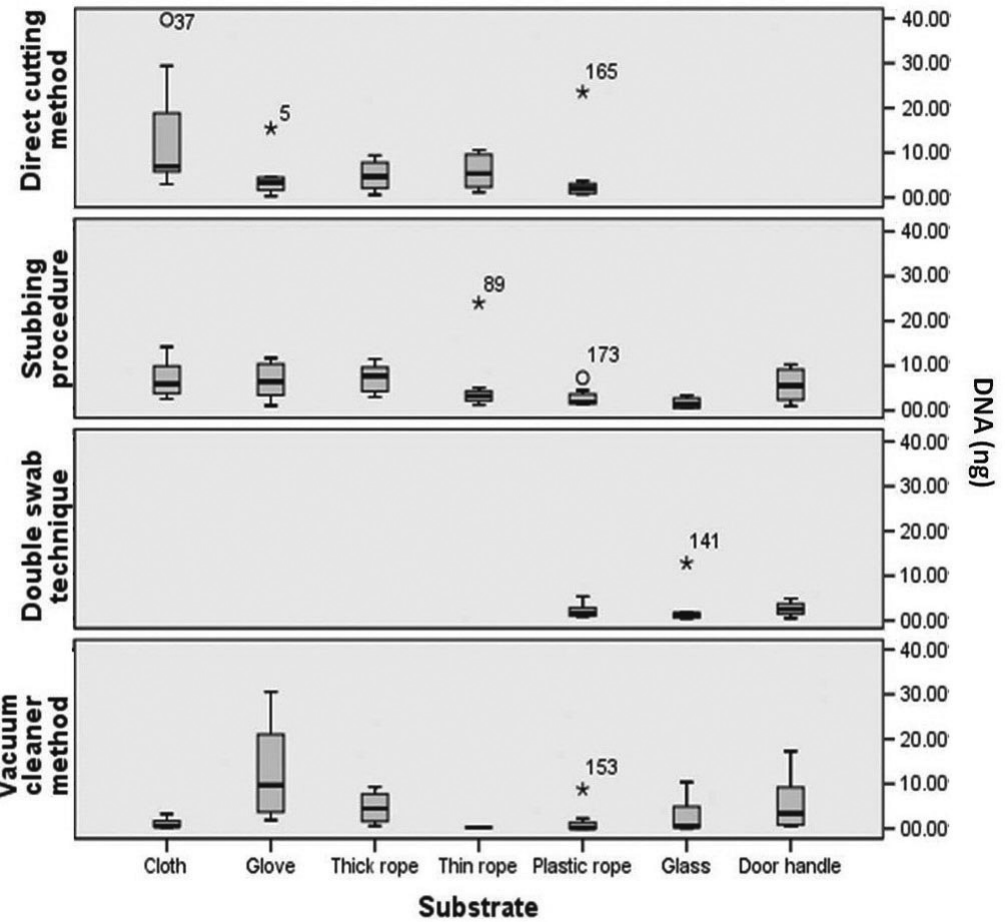
Amounts of DNA obtained by four different preprocessing methods: the direct cutting method, stubbing procedure, double swab technique, and vacuum cleaner method.

**Figure 2:**
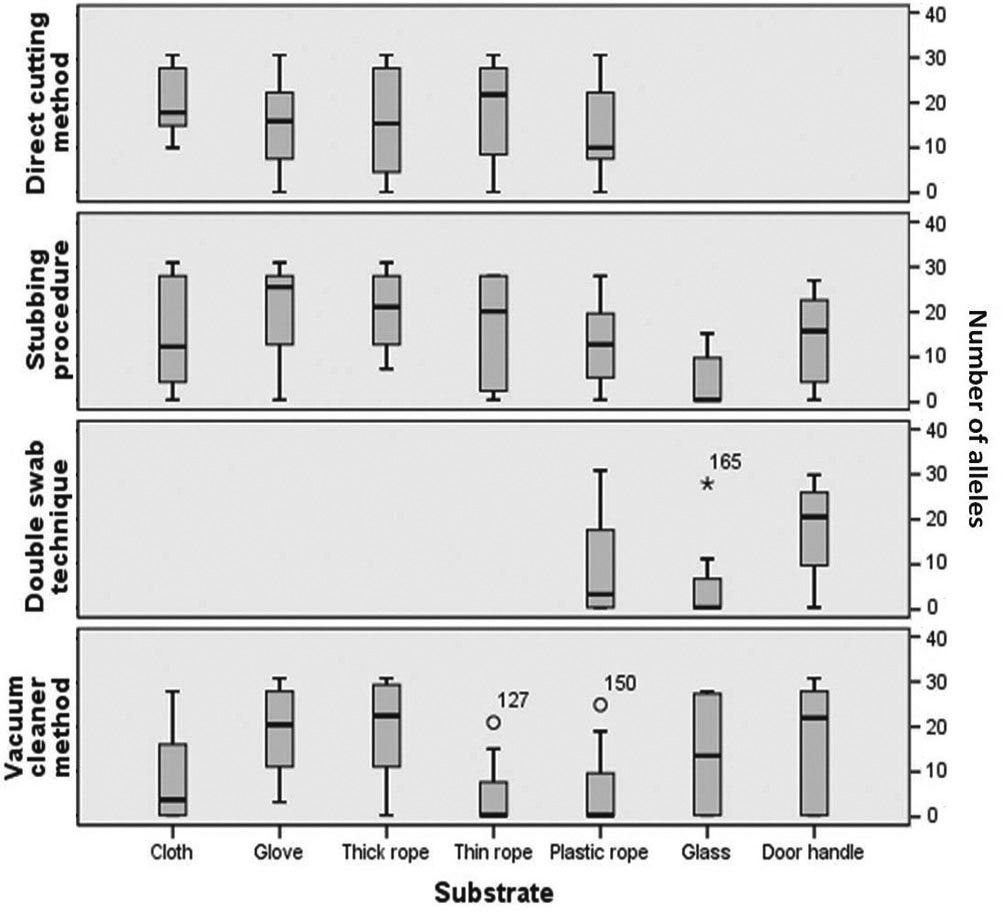
Alleles detected with four different preprocessing methods: the direct cutting method, stubbing procedure, double swab technique, and vacuum cleaner method.

The vast majority (95.5%) of the touch DNA samples contained detectable DNA. Among these samples, 100% of the porous substrate samples and 90% of the non-porous substrate samples (95.8% of the door handles, 79.2% of the glass, and 93.8% of the plastic ropes) contained detectable DNA. The average quality of total DNA from all samples and substrates analyzed was 4.54 ng. Various amounts of DNA were recovered from different substrates. The mean amount of DNA from porous substrates (5.93 ± 6.86 ng) was significantly higher than that from non-porous substrates (2.87 ± 4.13 ng; *P* < 0.001). DNA profiles were detected in all samples processed using the direct cutting method, 94.6% of samples processed using the stubbing procedure, 94.6% of samples processed using the vacuum cleaner method, and 91.7% of samples processed using the double swab technique. The detected amounts of DNA (Figure 1) and the number of alleles (Figure 2) differed based on the preprocessing method for each substrate. For the analysis of both DNA and alleles detected on cloth (F = 4.362, *P* = 0.026), thin cotton rope (F = 7.160, *P* = 0.004), and thick cotton rope (F = 4.116, *P* = 0.015), the direct cutting method demonstrated the best performance. However, for gloves, the vacuum cleaner method was better than the direct cutting method and the stubbing procedure (F = 4.713, *P* = 0.020). For door handles (F = 5.232, *P* = 0.014) and glass (F = 4.134, *P* = 0.031), the vacuum cleaner method was the best, and the direct cutting method was the best for plastic ropes (F = 3.256, *P* = 0.036) by a small margin.

**Figure 3:**
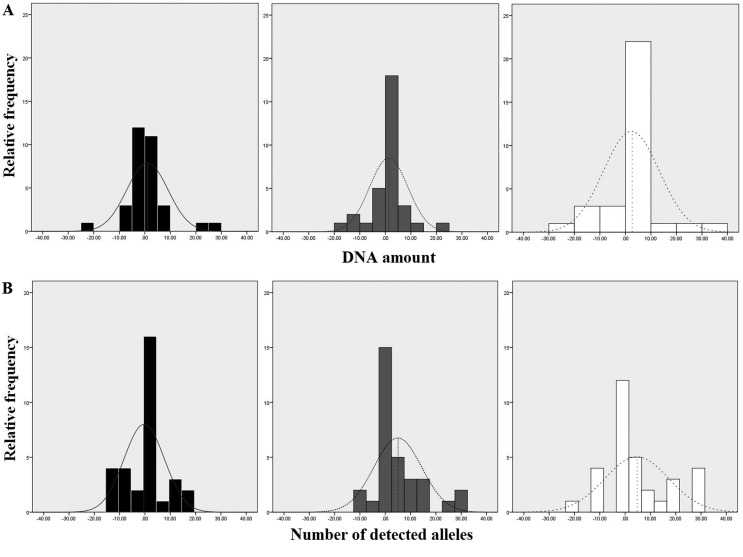
Differences in the amounts of DNA (**A**) and number of detected alleles (**B**) from the porous substrates among the three preprocessing methods: stubbing vs cutting (black bars); vacuum cleaner vs stubbing (gray bars), and vacuum cleaner vs cutting (white bars). The curves show a Gaussian distribution fit to the data. The maximums of the curves closer to 0, the more similar the two sets of data.

For the porous substrates, in order to directly compare the direct cutting method, the stubbing procedure and the vacuum cleaner method, the differences in the amount of DNA, and the number of detected alleles among three methods were determined. We subtract the results of the three sets of data from each other, and then used the subtracted numerical statistics to plot the frequency (Figure 3). The smaller the value of the subtraction, the smaller the difference between the data. The closer the maximums of the curves to 0, the more similar the two sets of data. The locations of the maximum of the curves reflect the differences between two preprocessing methods. Thus, the maximums of the curves of 1.0, 1.33, and 2.33 ng (Figure 3A) indicated that more DNA was obtained by using the direct cutting method and stubbing procedure than the vacuum cleaner method for the porous substrates (*P* < 0.001 for all). The maximums of the curves of -0.31, 4.94, and 4.63 alleles (Figure 3B) showed that more alleles were obtained by using the stubbing procedure than the vacuum cleaner method and by using the direct cutting method than the stubbing procedure (*P* < 0.001 for all).

**Figure 4:**
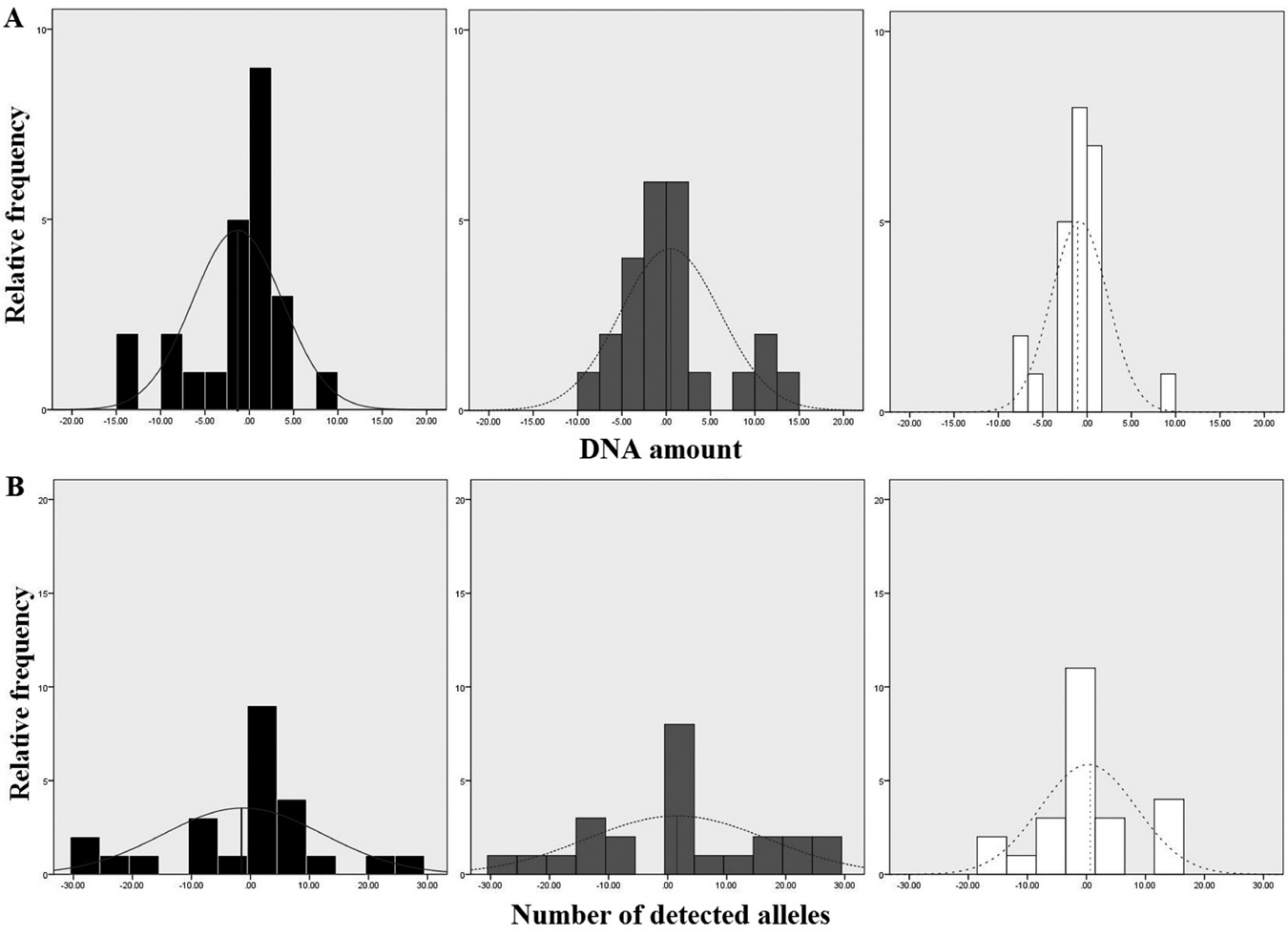
Differences in the amounts of DNA (**A**) and number of detected alleles (**B**) from the non-porous substrates among the three preprocessing methods: vacuum cleaner vs swab (black bars), stub vs vacuum cleaner (gray bars), and stub vs swab (white bars). The curves show a Gaussian distribution fit to the data.

We used four preprocessing methods to deal with non-porous substrates. Because the direct cutting method was only used with the plastic ropes, we compared the remaining three methods, ie, the vacuum cleaner, double swab, and stubbing, for preprocessing of non-porous substrates. For the non-porous substrates, the same analysis was performed (Figure 4). The maximums of the curves were -1.32, 0.46, and -0.86 ng (Figure 4A), showing that more DNA was obtained by using the vacuum cleaner method than the stubbing procedure and by using the stubbing procedure than the double swab technique (*P* < 0.001 for all). The maximums of the curves of -1.25, 1.58, and -0.33 alleles (Figure 4B) showed that more alleles were obtained by using the vacuum cleaner method than the stubbing procedure and by using the stubbing procedure than the double swab technique (*P* < 0.001 for all).

**Figure 5:**
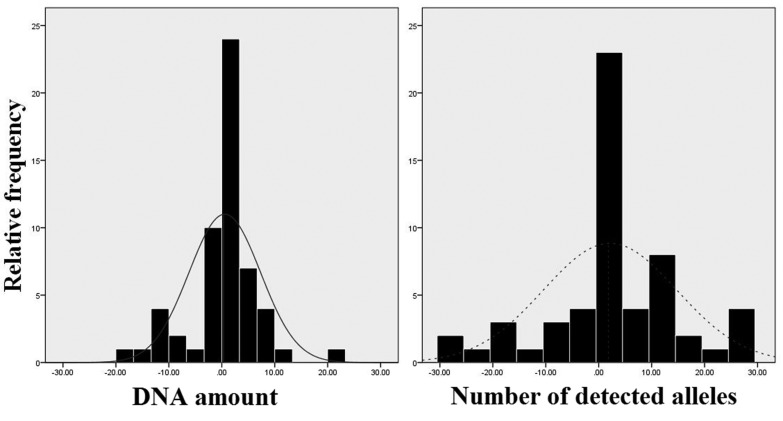
Differences in the amounts of DNA and the number of detected alleles between the stubbing procedure (left) and vacuum cleaner method (right) for all substrates. The curves show a Gaussian distribution fit to the data. The higher amounts of DNA and the number of detected alleles were obtained by using the stubbing procedure than the vacuum cleaner method.

In China, the direct cutting method and the double swab technique are the most frequently used methods for porous substrates and non-porous substrates, respectively. The stubbing procedure and the vacuum cleaner method are occasionally used to deal with both porous and non-porous substrate. Thus, the vacuum cleaner method and the stubbing procedure were further compared for all substrates (Figure 5). The maximums of the curves were 0.56 ng and 2.14 alleles, and the comparisons revealed that the vacuum cleaner method obtained less DNA and alleles than the stubbing procedure (*P* < 0.001 for both).

### Series B

**Figure 6:**
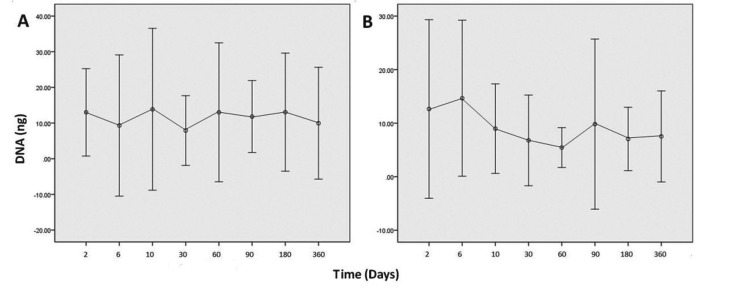
Average amounts of DNA remaining on cotton gloves (**A**) and door handles (**B**) after various storage time intervals. Each point represents the average of the five results for that particular storage time, and the error bars represent the standard error of the mean.

In series B, we used the stubbing procedure to deal with touch DNA samples after they had been stored for various lengths of time. DNA and alleles were detected in all 80 samples and all yielded full DNA profiles. Both the amounts of DNA and the number of alleles detected on the two types of substrates did not significantly change over 360 days of storage (F = 0.347, *P* = 0.926) (Figure 6). The highest and lowest amounts of DNA recovered from day 2 to day 360 from the cotton gloves were 26.98 and 2.44 ng, respectively (Figure 6A). The highest and lowest amounts of DNA recovered from day 2 to day 360 from the door handles were 23.21 and 2.58 ng, respectively (Figure 6B). Both the amounts of DNA recovered and the average peak heights of alleles detected did not decrease with time over 360 days (F = 1.577, *P* = 0.178).

## DISCUSSION

Through the comparison of DNA amount and allele numbers detected on porous substrates, the direct cutting method is more appropriate for cloth, thin cotton rope, and thick cotton rope; whereas the vacuum cleaner method is more appropriate for gloves. Through the comparison of DNA amount and allele numbers detected on non-porous substrates, the vacuum cleaner method is more appropriate for the door handles and glasses, and the direct cutting method is more appropriate for plastic ropes. The sizes of the clothes, thick cotton ropes, and thin cotton ropes were relatively smaller than the gloves, and they could be completely cut into pieces to extract DNA. For the glove, the vacuum cleaner method was able to sample the entire surface of the item, but only parts of the gloves were cut to extract DNA. This could explain the differences in the results for the preprocessing methods on gloves. Because the direct cutting method cannot be used on many non-porous substrates, and the other three methods in our experiment transfer DNA to tools for extraction, these transfer processes might result in the loss of some DNA. This may explain the good performance of the direct cutting method. It could reflect the fact that the differences were not just based on the substrates but also differences in how samples were handled and the amount of DNA placed on them.

In total, the amounts of DNA and the number of alleles detected on the porous substrates were greater than those on the non-porous substrates. For the porous substrates, the amounts of DNA and the number of alleles detected on the cotton gloves were the highest, and the thin cotton ropes yielded the least. For the non-porous substrates, the amounts of DNA and the number of alleles detected on the door handles were the greatest, and the glass yielded the least. When porous substrates, which are rough and contain pores, come into contact with skin or mucosa, epidermal cells can easily be deposited. However, the non-porous substrates were mostly smooth and less adherent or abrasive when in contact with skin or mucosa, providing less chance for epidermal cells to be left behind after contact (29). These physical characteristics of the porous and non-porous substrates may explain why more cells were found on the porous substrates and affected the final DNA typing results. The vacuum cleaner method was used with one-off tips. The membrane was used for extraction, but the tip may absorb some DNA and be ignored. The loss of DNA was obvious when dealing with touch DNA like on small cloth and rope samples. These results were different from the results with the gloves above because due to different conditions and comparisons. The non-porous substrates used in our experiment were smooth, it may not be easy for the swab or tape to collect cells or DNA from the surfaces of such substrates. However, the vacuum cleaner can easily collect cells or DNA from a smooth surface. Therefore, the vacuum cleaner method was the best for obtaining DNA from non-porous substrates. The vacuum cleaner method and the stubbing procedure were further compared for all substrates and the comparisons revealed that the vacuum cleaner method obtained less DNA and alleles than the stubbing procedure., which may be due to the loss of DNA on one-off tips.

The amounts of DNA extracted from the touch DNA samples were usually limited, which makes full profiles difficult to retrieve. The differences of the four preprocessing methods apparently affect the final DNA typing results. The direct cutting method and the double swab technique are simple and inexpensive. Additionally, these two preprocessing methods are well developed and were in use earlier than the other methods. Therefore, they are currently commonly used in case work. When using the vacuum cleaner method to deal with touch DNA samples, the quality of the samples is very important, and the vacuum cleaner tips were single-use. For the stubbing procedure, the EZ-tape was a one-off stub. The stubbing procedure and the vacuum cleaner method are more complicated and expensive than direct cutting and double swabbing. However, the usable range of the direct cutting method and the double swab technique are smaller than the stubbing procedure and the vacuum cleaner method, especially when dealing with samples with a large surface area. For example, we usually use the stubbing procedure and the vacuum cleaner method to deal with samples when the location of cells and/or DNA is difficult to determine.

We observed a different propensity of individual 5 to shed DNA, as reported previously (30,31), and perhaps provide some supporting examples. The eight individuals that prepared samples in this study were all in good health without skin diseases or metabolic disease. This phenomenon did not affect the reliability or authenticity of our experiment.

The cotton gloves were porous substrates, and the door handles were non-porous substrates. The five individuals that supplied the samples were all in good health without skin diseases or metabolic disease. To better observe the change of DNA after different storage times, 30 s was used to mock samples to get more DNA. Therefore, the amounts of DNA and the number of alleles detected were higher than that in series A. The samples were dry and stored in seal-lock plastic bags under arid conditions at ambient temperature (22-24°C). Because the samples were kept dry at room temperature, there was little DNA degradation over 360 days. This indicates that one should not ignore the value of samples that have been stored for many days.

In this study, we systematically investigated four preprocessing methods that are commonly used to deal with touch DNA samples, as well as the effects of storage time on touch DNA samples. We found that the amount of DNA and alleles detected on porous substrates were higher than on non-porous substrates. The direct cutting method was the best for porous substrates, and the vacuum cleaner method was the best for non-porous substrates. The amounts of DNA and the number and heights of the allele peaks detected on the two substrates over 360 days of storage displayed no significant downward trends. This study provides a theoretical basis for explorations of touch DNA samples and represents a reference when dealing with touch DNA samples in case work.

Further, this study is a part of a series of on-going investigations on touch samples in our laboratory. The results of this study have changed protocols at several DNA laboratories in China. Although different preprocessing methods have a significant impact on the detection of touch DNA samples, the choice of the extraction method after preprocess of the sample also plays a vital role in the examination of the sample. We believe that further studies will develop a more complete and better sample processing strategy based on the research achievements of this study.
